# Mycophenolate Mofetil Ameliorates Diabetic Nephropathy in db/db Mice

**DOI:** 10.1155/2015/301627

**Published:** 2015-08-04

**Authors:** Jung-Woo Seo, Yang Gyun Kim, Sang Ho Lee, Arah Lee, Dong-Jin Kim, Kyung-Hwan Jeong, Kyung Hye Lee, Seung Joon Hwang, Jong Shin Woo, Sung Jig Lim, Weon Kim, Ju-Young Moon

**Affiliations:** ^1^Division of Nephrology, Department of Internal Medicine, College of Medicine, Kyung Hee University, Seoul 134-727, Republic of Korea; ^2^Division of Cardiology, Department of Internal Medicine, College of Medicine, Kyung Hee University, Seoul 134-727, Republic of Korea; ^3^Department of Pathology, College of Medicine, Kyung Hee University, Seoul 134-727, Republic of Korea

## Abstract

Chronic low-grade inflammation is an important factor in the pathogenesis of diabetic complication. Mycophenolate mofetil (MMF) has an anti-inflammatory effect, inhibiting lymphocyte proliferation. Previous studies showed attenuation of diabetic nephropathy with MMF, but the underlying mechanisms were unclear. This study aimed to identify the effect of MMF on diabetic nephropathy and investigate its action mechanisms in type 2 diabetic mice model. Eight-week-old db/db and control mice (db/m mice) received vehicle or MMF at a dose of 30 mg/kg/day for 12 weeks. MMF-treated diabetic mice showed decreased albuminuria, attenuated mesangial expansion, and profibrotic mRNA expressions despite the high glucose level. The number of infiltrated CD4^+^ and CD8^+^ T cells in the kidney was significantly decreased in MMF-treated db/db mice and it resulted in attenuating elevated intrarenal TNF-*α* and IL-17. The renal chemokines expression and macrophages infiltration were also attenuated by MMF treatment. The decreased expression of glomerular nephrin and WT1 was recovered with MMF treatment. MMF prevented the progression of diabetic nephropathy in db/db mice independent of glycemic control. These results suggest that the effects of MMF in diabetic nephropathy are mediated by CD4^+^ T cell regulation and related cytokines.

## 1. Introduction

Diabetes mellitus (DM) is the most common cause of end-stage renal disease and the leading risk factor for cardiovascular disease. Current treatment for diabetes remains conservative care including glycemic and blood pressure control based on the renin-angiotensin system blockade with a low-protein diet and lipid-lowering agents. The therapeutic limitations originate from the incomplete understanding of the pathogenesis of diabetes complications. Recent studies have associated chronic inflammation with the development and aggravation of type 2 DM [1, 2]. In addition, some inflammatory factors such as tumor necrosis factor- (TNF-) *α*, interleukin- (IL-) 6, and IL-1*β* have been thought to predict macrovascular complications in type 2 DM [3, 4].

In particular, activation and infiltration of T lymphocytes with monocytes/macrophages in the kidney along with associated cytokines have been investigated in patients with diabetic nephropathy. We previously reported marked interstitial infiltration of CD4^+^ and CD8^+^ T cells with increased interferon- (IFN-) *γ* and TNF-*α* in streptozotocin- (STZ-) induced diabetic mice, as well as increased infiltration of CD4^+^, CD8^+^, and CD20^+^ cells in the renal interstitium of patients with type 2 diabetic nephropathy [5]. In addition, some results of previous investigations have indicated that mice deficient in intercellular adhesion molecule-1 had defects in leukocyte homing to the kidney, resulting in attenuation of renal injury [6]. Therefore, the recruitment of lymphocytes plays a key role not only in development but also in the progression of diabetic nephropathy.

Recently, mycophenolate mofetil (MMF) in experimental diabetic nephropathy was shown to ameliorate renal injury and prevent the development of nephropathy through various mechanisms [7–10]. Using MMF in diabetic nephropathy reduced various inflammatory cytokines and chemokines and attenuated podocyte apoptosis. Mycophenolic acid, the active form of MMF, inhibits T lymphocyte proliferation by blocking the early phases of the cell cycle and inducing apoptosis in stimulated T lymphocytes [11]. Based on these results, the inhibition of intrarenal T cells could possibly be the effect of MMF. However, there are only a few data regarding the effect of MMF in attenuating increased renal T cell infiltration in diabetic nephropathy. Previous diabetic animal study did not show a reduction in intrarenal T cell after MMF administration [7]. Furthermore, even though previous studies showed a podocyte-protective effect of MMF administration, the underlying mechanism has not been revealed.

The purpose of this study is to identify the role of MMF in diabetic nephropathy, especially in intrarenal T cell recruitment using db/db mice.

## 2. Materials and Methods

### 2.1. Animal Model and Experimental Design

Six-week-old male nondiabetic db/m and diabetic db/db mice were purchased from Jackson Laboratory (Sacramento, CA, USA). All mice received a diet of rodent pellets (348 kcal/100 g) containing 5.5% crude fat and tap water* ad libitum*. Mycophenolic acid was incorporated into chow (Dooyeol Biotech, Seoul, Republic of Korea) to reach an oral dose of 30 mg/kg body weight/day at a chow consumption of 0.2 g/g body weight. Various doses of MMF (20–40 mg/kg) were used in previous studies [7, 10, 12, 13]. In the preliminary study, we tested with a dose of 20 mg/kg/day, but there was no significant reduction in albuminuria in db/db group. At 8 weeks of age, mice were divided into three groups of six mice each: the nondiabetic control (db/m), the diabetic group (db/db), and the diabetes with MMF (Roche Pharma AG, Grenzach-Wyhlen, Germany) (db/db + MMF) from 8 to 20 weeks. During experiments, food intake, water intake, urine volume, body weight, fasting plasma glucose level, and glycosylated hemoglobin (HbA1c) were measured monthly. HbA1c was measured by immunoassay (DCA 2000 system; Bayer Diagnostics, Elkhart, IN, USA). To determine urinary albumin excretion, each individual mouse was placed in a metabolic cage and urine was collected for 24 hours. The urinary microalbumin concentration was determined by a competitive enzyme linked immunosorbent assay (ALPCO, NH, USA) and corrected by urinary creatinine (R&D Systems, Minneapolis, MN). All animal experiments were performed in compliance with the guidelines of the Animal Research Ethics Committee of Kyung Hee University, Seoul, Republic of Korea.

### 2.2. Light Microscopy

For light microscopy, the kidney tissue was fixed in 10% neutral buffered formalin, embedded in paraffin, cut into 3 *μ*m sections, and stained with periodic acid-Schiff (PAS) reagent. The degree of glomerular mesangial matrix expansion for each glomerulus was evaluated semiquantitatively using a score of 0–4: grade 0, no lesion; grade 1, <25%; grade 2, 25–50%; grade 3, 50–75%; and grade 4, <75%. At least 50 glomeruli per section were analyzed in a blinded manner.

### 2.3. Determination of Cytokines

Kidney tissues at 20 weeks were washed with phosphate-buffered saline and were homogenized on ice. Tissue homogenates were centrifuged to remove tissue residues at 12.000 g for 10 minutes at 4°C. Total protein concentration of the supernatant was measured using Pierce BCA protein assay kit. The cytokines were analyzed in the supernatant separated from tissue residues and plasma by using Luminex-bead array (Mouse Cytokine/Chemokine Magnetic Bead Panel, Millipore, Billerica, MA, USA) for detection of IFN-*γ*, TNF-*α*, IL-4, IL-6, and IL-17 according to the manufacturer's specifications. Concentrations of all cytokines measured were expressed as pg/mL.

### 2.4. Immunohistochemistry and TUNEL Assay

Immunohistochemistry for CD4 and CD8 was carried out using the Bond Polymer Refine Detection system (Vision BioSystems, Australia), according to the manufacturer's instructions with minor modifications. In brief, 4 *μ*m sections of formalin-fixed and paraffin-embedded tissues were deparaffinized by Bond Dewax Solution and an antigen retrieval procedure was carried out using Bond ER solution for 30 min at 100°C. Endogenous peroxidase was quenched by incubation with hydrogen peroxide for 5 min. Sections were incubated with primary polyclonal antibodies for CD4 (1 : 100, Abcam, Cambridge, UK) and CD8 (1 : 100) using the biotin-free polymeric horseradish peroxidase-linked antibody conjugate system in a Bond-maX automatic slide stainer (Vision BioSystems, USA).

Immunohistochemical staining of CD68, for macrophages, and nephrin and Wilms tumor-1, for podocytes, was performed manually. Deparaffinized sections were rehydrated and microwaved in 10 mM citrate buffer (pH 6.0) for 20 minutes. After the retrieval, the sections were incubated in 3% H_2_O_2_ in methanol for 30 minutes to block peroxidase activity. The sections were then washed in PBST, blocked with 1% BSA/PBS for 30 minutes, and incubated with the primary antibodies: CD68 (1 : 100, AbD Serotec, NC, USA); nephrin (1 : 100, ENZO Life Sciences, NY, USA); and WT1 (1 : 100, Santa Cruz, CA, USA). For visualization the ZytoChem Plus HRP Polymer Kit (Zytomed Systems, Berlin, Germany) and the 3,3′-diaminobenzidine (DAB) substrate (Bethyl Laboratory, TX, USA) were used. Counterstaining was performed with hematoxylin except WT1. Infiltrated lymphocytes and macrophages were counted in 10 interstitial fields and 20 glomeruli per mouse under ×200 and ×400 magnification. Nephrin-positive area was measured in 30 glomeruli per mouse using ImageJ software, version 1.49o (NIH, Bethesda, USA). WT1-positive cells were counted in at least 30 randomly selected glomeruli under ×400 magnification.

For tubular and glomerular apoptotic scores, In Situ Cell Death Detection Kit, Fluorescein (Roche Applied Science, USA), was used according to the manufacturer's instruction. TUNEL-positive cells were screened, counted, and analyzed using a fluorescence microscope in 10 interstitial fields and 20 glomeruli per kidney.

### 2.5. Isolation of Total RNA, RT, and Real-Time PCR

Total RNA was extracted from kidney tissue using the Total RNA Isolation Kit (MACHEREY-NAGEL, Germany). Real-time PCR was performed using SYBR Green PCR Master Mix (FastStart Universal SYBR Green Master, Roche). The real-time PCR reaction was performed with an ABI StepOne real-time PCR system (Applied Biosystems, USA) following the manufacturer's guidelines. The primers for transforming growth factor-*β*1 (TGF-*β*1), connective tissue growth factor (CTGF), type I collagen, type IV collagen, CXCL1 (chemokine (C-X-C motif) ligand 1), CXCL2, CXCL9, CCL (chemokine (C-C motif) ligand) 2, CCL3, CCL20, and 18S are used. The sequences of used primers are shown in the supplementary Table 1 in the Supplementary Material available online at  http://dx.doi.org/10.1155/2015/301627. Each sample was run in triplicate in separate tubes for reproducibility. The target gene expression levels were normalized to 18S expression.

### 2.6. Western Blot Analysis

Kidney tissues were washed with phosphate-buffered saline and lysed with ice cold lysis buffer (10 mMTris·HCl, 150 mMNaCl, 1% Triton X-100, 5 mM EDTA pH 8.0) and protease inhibitor cocktail (Roche Diagnostics, Mannheim, Germany). The lysate was centrifuged at 4°C for 10 min at 10,000 g and the supernatant was recovered. Equal amounts of total cellular protein were subjected to SDS/PAGE in 12% acrylamide gel and then transferred to a PVDF membrane (Millipore, Madrid, Spain) by electroblotting, and the membrane was blocked with 5% fat-free milk in Tris-buffered saline with 0.5% Tween 20 (TBS-T). The membranes were incubated with primary antibodies against *β*-actin, NADPH oxidase 4 (Nox4), p67-phox (1 : 1000, Santa Cruz, CA, USA), Bax, Bcl-2 (1 : 1000, Cell Signaling Technology, MA, USA) in TBS-T, and 5% Bovine Serum Albumin (BSA) overnight at 4°C. Then the blots were washed and incubated with secondary antibody in blocking solution (goat anti-rabbit horseradish peroxidase- (HRP-) conjugated and goat anti-mouse HRP-conjugated, 1 : 10000, Santa Cruz, CA, USA) for 2 h at room temperature. The signal was detected by a pico enhanced peroxidase detection (EPD) western blot detection kit (Mbiotech, Seoul, Republic of Korea), and bands were visualized using a G: Box chemi XL (Syngene, Cambridge, UK). *β*-actin was used as an internal control.

### 2.7. Statistical Analysis

All values are expressed as means ± SE. Results were analyzed using the Kruskal-Wallis nonparametric test for multiple comparisons. Significant differences in the Kruskal-Wallis test were confirmed by the Wilcoxon rank sum, Mann-Whitney, and Friedman test (used to compare mean differences); *p* values < 0.05 were considered statistically significant.

## 3. Results

### 3.1. MMF Treatment Attenuates Diabetic Nephropathy Independent of Glycemic Control

**Figure 1:**
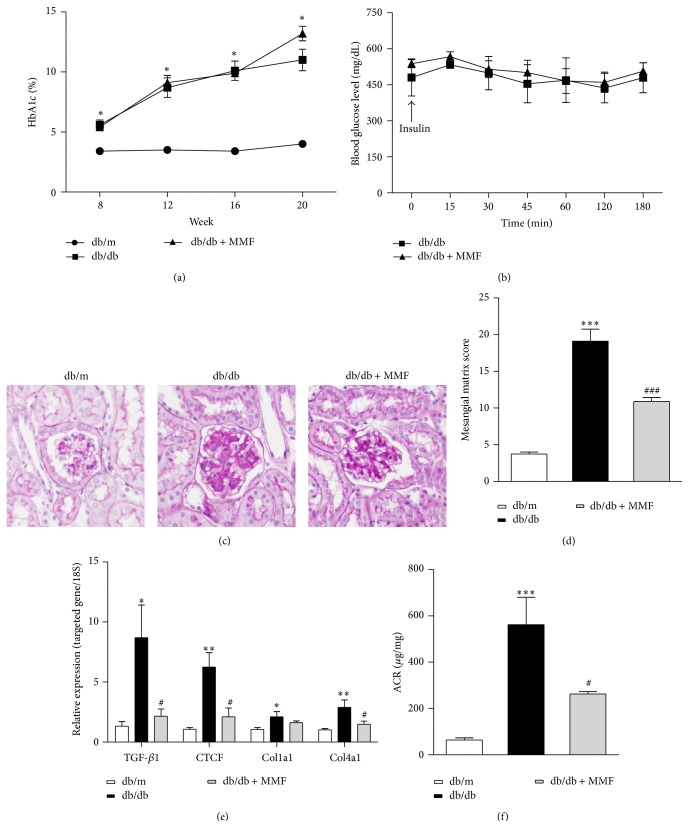
The effect of MMF on glucose control and diabetic nephropathy of db/db mice. HbA1c and insulin tolerance test (a and b). Kidney injury was observed by PAS staining (original magnification ×400) (c), and the glomerular matrix score was quantified (d). Real-time PCR for TGF-*β*1, CTGF, and type I and type IV collagen (e). Urinary albumin/creatinine excretion (f). The results are expressed as mean ± SE. ^*∗*^ *p* < 0.05, ^*∗∗*^ *p* < 0.01, ^*∗∗∗*^ *p* < 0.001 versus db/m; ^#^ *p* < 0.05, ^###^ *p* < 0.001 versus db/db.

The 30 mg/kg dose of MPA was well-tolerated, and there were no typical side effects such as diarrhea and weight loss. The various biochemical parameters were determined in three experimental groups of mice, as shown in Table 1. Both db/db and db/db + MMF mice showed higher HbA1c level than db/m mice; in particular, there was no difference in HbA1c between db/db and db/db + MMF mice at 20 weeks (Figure 1(a)). The result of insulin tolerance test also showed no difference between db/db and db/db + MMF mice (Figure 1(b)). To determine the severity of diabetic renal injury in each group, we confirmed the mesangial matrix expansion by PAS staining. As shown in Figures 1(c) and 1(d), MMF treatment improved mesangial matrix expansion in the db/db mouse kidney. In addition, MMF treatment reduced TGF-*β*1, CTGF, type I collagen, and type IV collagen mRNA expression in the diabetic mice kidney (Figure 1(e)). The increased urinary albumin/creatinine excretion ratio was significantly decreased by MMF treatment (Figure 1(f)). These findings suggest that MMF has renoprotective effect in diabetic nephropathy without glycemic control.

### 3.2. MMF Decreases T Lymphocyte Infiltration and Its Related Cytokines in the db/db Kidney

**Figure 2:**
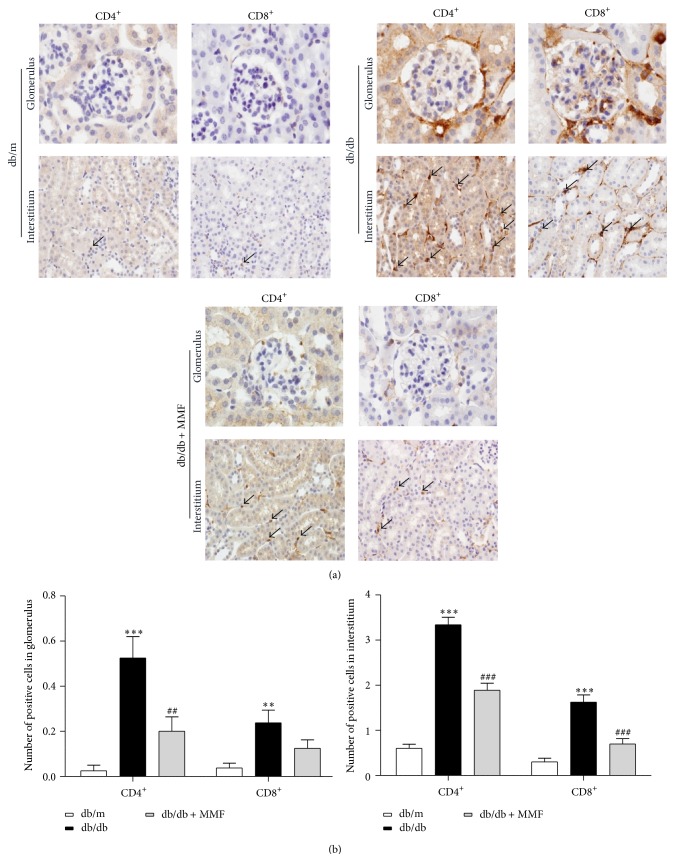
MMF attenuates T cells infiltration in diabetic kidney. Infiltration of renal CD4^+^ and CD8^+^ cells in glomeruli and tubulointerstitium was observed by immunohistochemical analysis (a) and the infiltration of CD4^+^ and CD8^+^ cells was quantitatively scored (b). Original magnification ×400. The results are expressed as the means ± SE. ^*∗∗*^ *p* < 0.01, ^*∗∗∗*^ *p* < 0.001 versus db/m; ^##^ *p* < 0.01, ^###^ *p* < 0.001 versus db/db.

**Figure 3:**
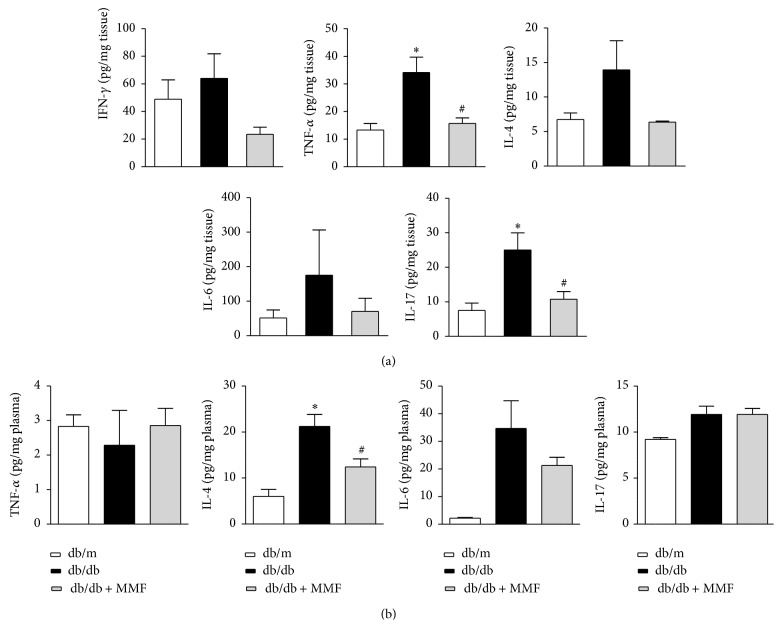
MMF decreases CD4^+^ T cells related cytokines in the db/db kidney. The cytokines IFN-*γ*, IL-4, IL-6, IL-17, and TNF-*α* were detected by a Luminex-bead array in kidney (a) and plasma (b). The results are expressed as mean ± SE. ^*∗*^ *p* < 0.05 versus db/m; ^#^ *p* < 0.05 versus db/db.

As shown in Figure 2, infiltration of CD4^+^ and CD8^+^ T cells in tubulointerstitium was significantly increased in db/db mice compared with db/m mice. MMF treatment markedly reduced the number of infiltrated CD4^+^ and CD8^+^ T cells in the db/db kidney. To confirm the effect of MMF on regulating intrarenal T cells, we investigated systemic and intrarenal T cell-related cytokines which are known to regulate inflammatory and immune responses to the development of progression of diabetic nephropathy. MMF treatment significantly decreased intrarenal TNF-*α* and IL-17 levels (Figure 3(a)). The plasma levels of IL-4 and IL-6 were increased in db/db mice and were decreased by MMF treatment. The increased levels of TNF-*α* and IL17a in db/db were reduced due to MMF treatment in the kidneys, but no changes were observed in plasma.

### 3.3. MMF Reduces Renal Chemokines and Macrophages Recruitment

**Figure 4:**
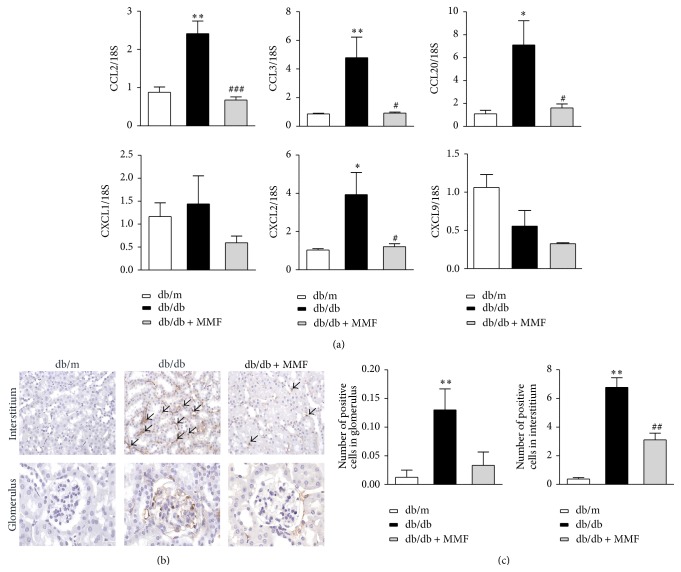
MMF reduces renal chemokines and macrophages recruitment. Real-time PCR for CCL2, CCL3, CCL20, CXCL1, CXCL2, CXCL9, and 18S (a). Infiltrating the renal CD68^+^ cells in glomeruli and tubulointerstitium was observed by immunohistochemical analysis (b) and the infiltration of CD68^+^ cells was quantitatively scored (c). Each sample was run in triplicate in separate tubes to permit quantification of the target gene expression normalized to 18S expression. The results are expressed as mean ± SE. ^*∗*^ *p* < 0.05, ^*∗∗*^ *p* < 0.01 versus db/m; ^#^ *p* < 0.05, ^##^ *p* < 0.01, and ^###^ *p* < 0.001 versus db/db.

Next, we examined the expression of intrarenal chemokines associated with CD4+ T cells. The expression of CCL2, CCL3, and CCL20, which are controlled by IL-17, was significantly increased and attenuated by MMF treatment in the db/db mouse kidney (Figure 4(a)). The number of infiltrated CD68+ macrophages in tubulointerstitial area in db/db mice was significantly increased compared with db/m mice but was also markedly reduced by MMF treatment (Figures 4(b) and 4(c)). These results indicate that MMF treatment attenuates diabetic nephropathy by decreasing macrophage infiltration in the kidney through the regulation of IL-17-related chemokines in db/db mice.

### 3.4. MMF Attenuates Podocyte Injury

**Figure 5:**
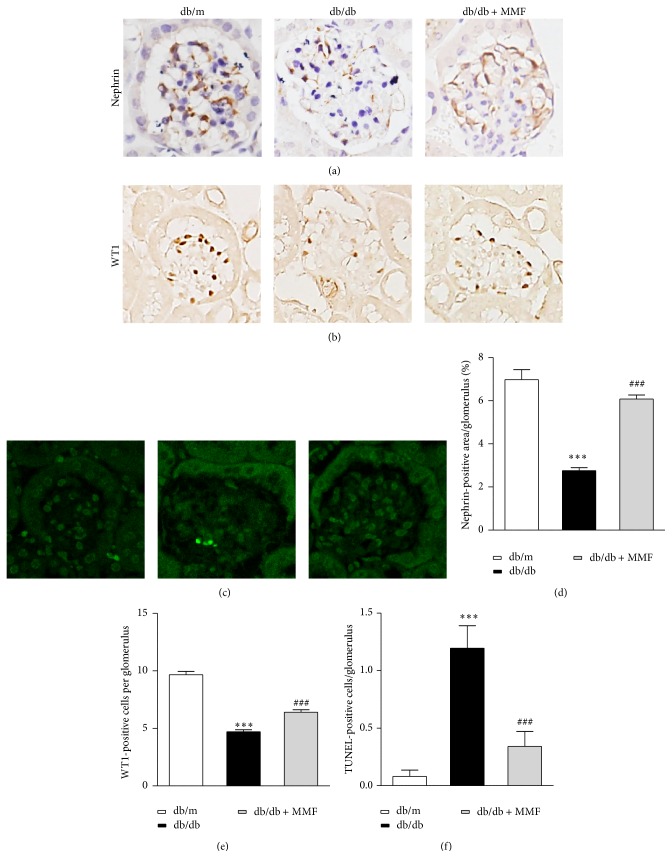
The effect of MMF on podocyte injury. Nephrin and WT1 are represented for db/m, db/db, and MMF-treated db/db mice (a, b and d, e, resp.). TUNEL assay for apoptotic injury in glomeruli (c), and TUNEL-positive cells counted for quantitative analysis (f) in three experimental groups. The results are expressed as mean ± SE. ^*∗∗∗*^ *p* < 0.001 versus db/m; ^###^ *p* < 0.001 versus db/db. Original magnification ×400.

We investigated podocyte injury to examine the role of MMF in the diabetic kidney related with improved microalbuminuria. The loss of glomerular nephrin was recovered in MMF-treated db/db mice compared with db/db mice (Figures 5(a) and 5(d)). WT1 was also increased by MMF treatment (Figures 5(b) and 5(e)). TUNEL-positive apoptotic cells in glomeruli were increased in db/db mice and were attenuated by MMF treatment (Figures 5(c) and 5(f)). These findings indicated that MMF attenuates diabetic nephropathy by reducing podocyte apoptosis.

### 3.5. The Effects of MMF on Apoptosis and ROS in the Diabetic Kidney

**Figure 6:**
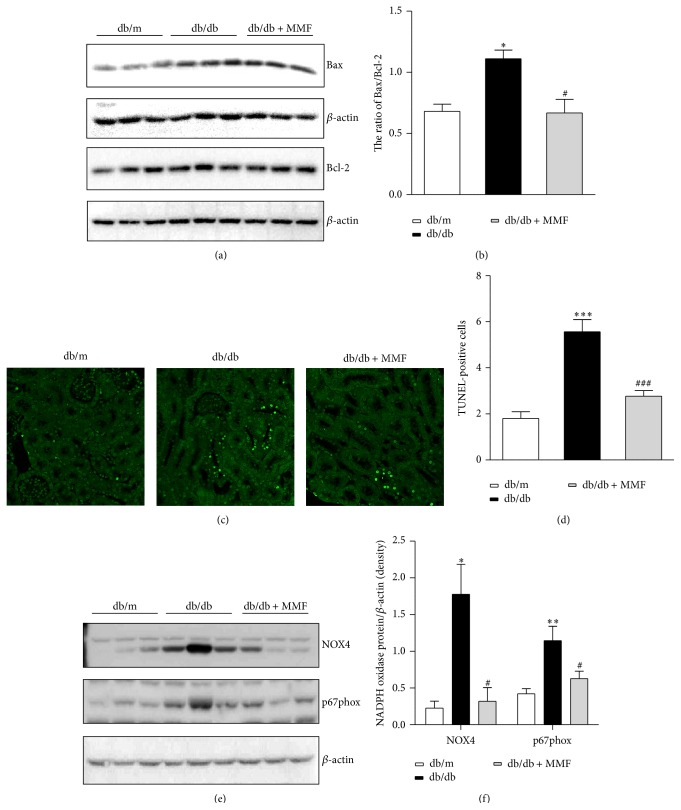
The effect of MMF on apoptotic injury and NAD(P)H oxidase. The proapoptotic Bax and antiapoptotic Bcl2 were analyzed by western blotting (a) and quantitative analyses of the Bax/Bcl2 ratio (b). Tubulointerstitial TUNEL staining (c) and TUNEL-positive cells were assessed for quantitative analysis (d) in three experimental groups. Original magnification ×400. Representative western blot images of NADPH oxidase, NOX4, and p67phox are shown (e). The quantification of NADPH oxidase (f). The results are expressed as mean ± SE. ^*∗*^ *p* < 0.05, ^*∗∗∗*^ *p* < 0.001 versus db/m; ^#^ *p* < 0.05, ^###^ *p* < 0.001 versus db/db.

We also evaluated the effect of MMF on apoptotic injury in renal tubules of db/db mice. The Bax/Bcl-2 ratio in the diabetic kidney was decreased with MMF treatment (Figures 6(a) and 6(b)). TUNEL-positive apoptotic cells in the tubular area were also attenuated by MMF treatment (Figures 6(c) and 6(d)). In addition, we evaluated the effect of MMF on diabetic ROS injury in the kidney. Increased expression of Nox4 and p67phox in the kidney of db/db mice was decreased by MMF (Figures 6(e) and 6(f)).

## 4. Discussion

Results from this study indicate that MMF treatment ameliorates diabetic nephropathy in db/db mice with a decrease in albuminuria, mesangial proliferation, and podocyte injury regardless of hyperglycemia. This study shows that MMF decreases intrarenal CD4^+^ and CD8^+^ T cell recruitment in db/db mice. Previous study showed that MMF administration in STZ-induced diabetic rats did not result in reduction in CD3^+^ lymphocyte infiltration in the renal cortex [7]. Sequentially, T cell inhibition led to reduced expression of T cell-related cytokines and chemokines. These anti-inflammatory effects of MMF contributed to improvement in diabetic nephropathy.

Diabetic complications, especially diabetic nephropathy, were ameliorated by intensive blood glucose and blood pressure control. Glucose control is very important factor to reduce microvascular complications. Unfortunately, many diabetic nephropathy patients progress to end-stage renal disease despite the use of strict glucose and blood pressure control, and these subgroups of diabetic nephropathy patients may be susceptible to inflammatory injury. Although HbA1c was not reduced, the beneficial effects of MMF on diabetic nephropathy were found in this study. These results indicate that a partial role of immune cells-related inflammatory injury is one of the important therapeutic targets to diabetic nephropathy.

Increased CD4^+^ and CD8^+^ T cell infiltration was attenuated in the interstitium after 12 weeks of MMF treatment in db/db mice. When we measured intrarenal Th1- and Th17-associated cytokines, we confirmed that IL-17 and TNF-*α* were decreased by MMF treatment. Particularly, IL-17-associated chemokines such as CCL2, CCL3, and CCL20 [14] were significantly attenuated.

An interesting finding is that the increased T cell-related cytokines in plasma were different to those in the kidney. The plasma levels of IL-4 and IL-6 were increased in db/db mice and were decreased by MMF treatment. The levels of TNF-*α* and IL-17 were reduced due to MMF treatment in the kidneys, but no changes were observed in plasma. A previous study also reported that MMF treatment reduced T cells in local atherosclerotic inflammatory region, not systemic T cells [13]. Therefore, we carefully conclude that systemic inflammatory activity is different from that in the kidney and that the renal T cells are more susceptible to MMF treatment in db/db mice. These results suggest that the diabetic kidney with albuminuria is more actively responsive to inflammation than systemic circulation.

Th1 response is recognized as accompanying type 1 diabetes [15], and enhanced Th1-related cytokines/chemokines have been correlated with proteinuria and renal function in type 2 diabetes [16]. MMF treatment might contribute to reduced proteinuria and improved pathologic findings by reducing renal CD4^+^ T cell and aberrantly regulating its cytokines/chemokines. An interesting finding in this study is that MMF more sensitively regulated intrarenal IL-17 which is related to CCL2, CCL3, and CCL20. Increase in the number of Th17 cells is considered as a part of the pathogenesis of diabetes, and it has been supported by findings in type 1 DM murine model and by human data [17, 18]. Although a precise relationship between IL-17 and diabetic nephropathy needs to be confirmed, our results suggest that IL-17 could play proinflammatory role in diabetic nephropathy and that MMF has beneficial effects in reducing intrarenal IL-17 and related chemokine levels. In addition, the levels of CXCL2, which is known to arrest rolling monocytes [19], were attenuated in the kidney of MMF-treated db/db mice. Therefore, the suppression of intrarenal immune cells by MMF might lessen the secretion of proinflammatory cytokines/chemokines, and the downregulation of these cytokine/chemokines could attenuate macrophage recruitment.

Nephrin, a slit diaphragm-associated protein, prevents the development of albuminuria, and WT1, a podocyte-specific marker, reflects podocyte number [20, 21]. In diabetic nephropathy, podocytes are important in regulating glomerular filtration, and the loss of podocytes leads to glomerular damage and causes albuminuria. In the current work, MMF treatment preserved podocytes in db/db mice. Although the exact mechanisms associated with podocyte apoptosis are still unknown, a previous work has shown that TGF-*β*1 induces apoptosis in cultured podocytes [22]. Another study demonstrated that high glucose induces reactive oxygen species (ROS) generation and leads to apoptosis of podocytes [21]. Treatment with NADPH oxidase inhibitor alleviates podocyte apoptosis and prevents glomerular injury in type 1 and type 2 diabetic animal models [23]. Therefore, the beneficial role of MMF in protecting against the loss of podocytes is thought to arise from its antioxidant and anti-inflammatory effects.

ROS and apoptosis are important mechanisms of diabetic nephropathy which is largely dependent on glycemic control. Upregulated Bax and downregulated Bcl-2 expression were reversed by MMF treatment, as was also confirmed by TUNEL staining. We also found that through NAD(P)H oxidase, a critical generator of ROS [24], MMF could be associated with the attenuation of ROS injury independent of hyperglycemia. In db/db mice, Nox4 induces ROS generation and regulates profibrotic renal injury through p38 mitogen-activated protein kinase [25]. However, our results indicated that MMF attenuated intrarenal Nox4 and p67phox regardless of high blood glucose level. Proinflammatory immune responses promoted by diabetes are associated with augmented ROS generation by NAD(P)H oxidase [26], and inflammatory cytokines contribute to activation of ROS injury through upregulation of NAD(P)H oxidase and inducible NO synthase [27].

In conclusion, we demonstrated that MMF treatment attenuates diabetic nephropathy by decreasing CD4^+^ T cell infiltration and its related cytokines and chemokines. The anti-inflammatory effect of MMF attenuates podocyte apoptosis independent of glucose control. This result suggests that MMF may have a beneficial role in the treatment of diabetic nephropathy.

## Supplementary Material

Supplementary Table 1: List of mouse primers forward primer (F) and reverse primer (R) used for amplification using real-time PCR.

## Figures and Tables

**Table 1:** The clinical parameters of study groups.

Characteristics	Week	db/m	db/db	db/db + MMF
Body weight (g)	8 20	27.7 ± 0.25 31.4 ± 0.5	42.9 ± 1.76^*∗*^ 51.0 ± 3.2^*∗*^	45.2 ± 0.37^*∗*^ 50.1 ± 1.9^*∗*^
Daily food intake (g/day)	8 20	3.97 ± 0.01 5.6 ± 0.4	5.82 ± 0.28^*∗*^ 7.6 ± 1.2^*∗*^	5.76 ± 0.17^*∗*^ 8.8 ± 0.8^*∗*^
Daily water intake (mL/day)	20	5.0 ± 0.3	13.5 ± 0.6	15.6 ± 1.6
Urine output (mL/day)	20	0.4 ± 0.2	2.4 ± 1.4^*∗*^	7.5 ± 3^*∗*#^
Fasting glucose (mg/dL)	20	95.8 ± 14.4	391 ± 40.6^*∗*^	434.9 ± 40.7^*∗*^
Kidney/BW (%)	20	1.54 ± 0.04	1.33 ± 0.17	1.05 ± 0.02^*∗*^
Liver/BW (%)	20	4.85 ± 0.65	5.9 ± 0.33	6.1 ± 0.38
Fat/BW (%)	20	3.2 ± 0.5	4.9 ± 0.17	6.3 ± 0.6^*∗*^

MMF: mycophenolate mofetil.

The results are expressed as the mean ± S.E.M.

^*∗*^Significantly different with respect to the db/m mice;  ^#^significantly different with respect to db/db mice.

^*∗*#^ *p* < 0.05.
