# Supplementary material for: Mycophenolate Mofetil Ameliorates Diabetic Nephropathy in db/db Mice
Source: Biomed Res Int. 2015 Aug 4;2015:301627. doi: 10.1155/2015/301627 (PMC4539432; doi:10.1155/2015/301627)
Supplement: Supplementary file 1 — Supplementary Table 1: List of mouse primers forward primer (F) and reverse primer (R) used for amplification using real-time PCR. [file 301627.f1.pdf]

Supplementary table 1. List of mouse primers forward primer (F) and reverse primer (R) used for amplification using real-time PCR.

Gene Name	Primer sequence
CCL2	F: 5'-TTAAAAACCTGGATCGGAACCAA-3'<br>R: 5'-GCATTAGCTTCAGATTTACGGGT-3'
CCL3	F: 5'-TGTACCATGACACTCTGCAAC-3'<br>R: 5'-CAACGATGAATTGGCGTGGAA-3'
CCL20	F: 5'-ACTGTTGCCTCTCGTACATACA-3'<br>R: 5'-GAGGAGGTTCACAGCCCTTTT-3'
CXCL1	F: 5'-CTGGGATTCACCTCAAGAACATC-3'<br>R: 5'-CAGGGTCAAGGCAAGCCTC-3'
CXCL2	F: 5'-AGGGCGGTCAAAAAGTTTGC-3'<br>R: 5'-CAGGTACGATCCAGGCTTCC-3'
CXCL9	F: 5'-CGAGGCACGATCCACTACAA-3'<br>R: 5'-GAGTCCGGATCTAGGCAGGT-3'
18S rRNA	F: 5'-GTAACCCGTTGAACCCCATT-3'<br>R: 5'-CCATCCAATCGGTAGTAGCG-3'
